# Proof-of-concept for using machine learning to facilitate data extraction for human health chemical assessments: a study protocol

**DOI:** 10.1080/2833373x.2024.2421192

**Published:** 2024-11-11

**Authors:** Michelle Angrish, Kristina A. Thayer, Brittany Schulz, Artur Nowak, Amanda Persad, Allison L. Phillips, Glenn Rice, Teresa Shannon, A. Amina Wilkins, Krista Christensen, Elizabeth G. Radke, Andrew Shapiro, Michele M. Taylor, Vickie R. Walker, Andrew A. Rooney, Sean M. Watford

**Affiliations:** a Center for Public Health and Environmental Assessment, Chemical and Pollutant Assessment Division, US Environmental Protection Agency, Durham, NC, USA; b Environmental Protection Agency National Student Services Contract, Oak Ridge Associated Universities, Oak Ridge, TN, USA; c Evidence Prime Inc, Hamilton, Canada; d Center for Public Health and Environmental Assessment, Chemical and Pollutant Assessment Division, US Environmental Protection Agency, Cincinnati, OH, USA; e Center for Public Health and Environmental Assessment, Health and Environmental Effects Assessment Division, US Environmental Protection Agency, Washington, DC, USA; f Division of Translational Toxicology, National Institute of Environmental Health Sciences, Durham, NC, USA

**Keywords:** Artificial intelligence, machine learning, systematic evidence map, systematic review, risk assessment

## Abstract

**Background:**

Systematic review (SR) methods are relied upon to develop transparent, unbiased, and standardized human health chemical assessments. The expectation is that these assessments will have discovered and evaluated all of the available information in a trackable, transparent, and reproducible manner inherent to SR principles. The challenge is that chemical assessment development relies on mostly literature-based data using manual approaches that are not scalable. Various SR tools have increased the efficiency of assessment development by implementing semi-automated approaches (human in the loop) for data discovery (literature search and screening) and enhanced data repositories with standardized data collection and curation frameworks. Yet filling these repositories with data extractions has remained a manual process and connecting the various tools together in one interoperable workflow remains challenging.

**Objectives:**

The objective of this protocol is to explore incorporation of a semi-automated data extraction tool (Dextr) into a chemical assessment workflow and understand if the new tool improves overall user experience.

**Methods:**

The workflow will use template systematic evidence map (SEM) methods developed by the Environmental Protection Agency for the identification of included studies. The methods described focus on the data extraction component of the workflow using a fully manual or a semi-automated (human in the loop) data extraction approach. Both the manual and semi-automated data extractions will occur in Dextr. The new data extraction tool will be evaluated for user experience and whether the data extracted using the automated approach meets or exceeds metrics (precision, recall, and F1 score) for a fully manual data extraction.

**Discussion:**

Artificial intelligence (AI) and machine learning (ML) methods have rapidly advanced and show promise in achieving operational efficiencies in chemical assessment workflows by supporting automated or semi-automated SR methods, possibly improving the user experience. Yet incorporating advances into sustainable workflows has remained a challenge. Whether using a tool like Dextr improves operational efficiencies and the user experience remains to be determined.

## 1. Introduction

**Figure 1.**
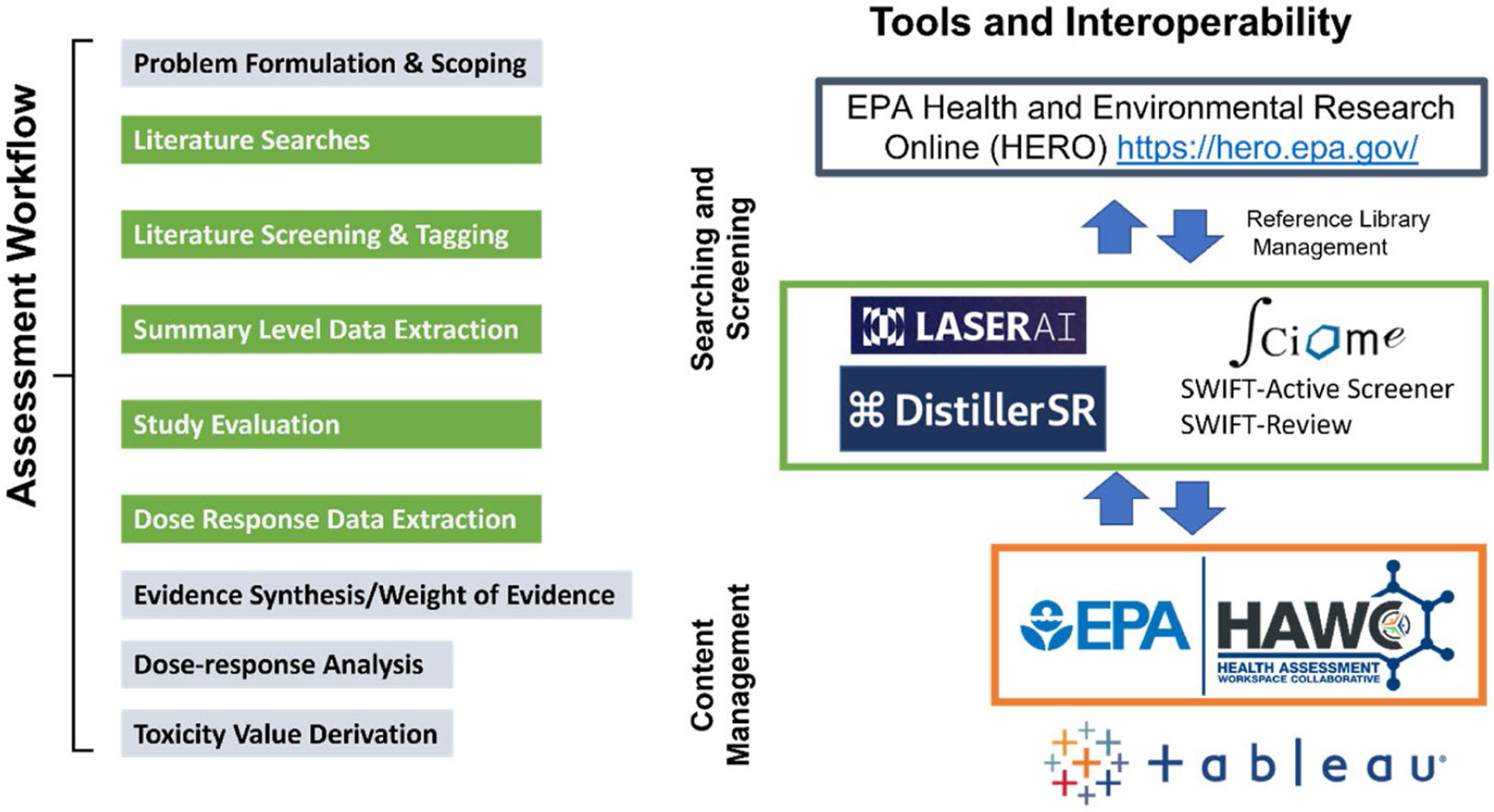
The workflow for conducting a chemical assessment is described on the left. The tools listed on the right support modules in that workflow, particularly those colored in the green boxes. EPA's Health and Environmental Research Online (HERO) facilitates literatures searches and reference library management. Evidence Partner's DistillerSR, and Sciome's SWIFT-Active Screener and SWIFT-Review are useful literature screening and tagging tools. DistillerSR is also used for data extraction supporting SEM development. EPA Health Assessment Workplace Collaborative (HAWC) is the primary content management system for chemical assessments and facilitates reference, management, screening, literature tagging, data extraction and visualization. Tableau is typically used to make available SEM visuals.

The field of chemical assessment is an essential component of public health, aimed at evaluating the hazards associated with environmental exposure for the purpose of protecting human and environmental health. The Environmental Protection Agency's Chemical Pollutant Assessment Division (CPAD) is tasked with developing various human health assessments that evaluate all of the available information in a manner that is trackable, transparent, and unbiased. CPAD has made significant advancements in assessment development processes by implementing standardized workflows and tools that increase the efficiency of information review and management (U.S. EPA 2022) (Figure 1). A major challenge to assessment development has been scaling traditional assessment approaches to meet the rate that information is made available and organizing/sorting/labeling that information once it has been found. For example, PubMed covers approximately 26,000 journals and contains approximately 36 million citations (accessed March 27, 2024).

Advantageously, as technological advances have improved access to information, they have also advanced the methods, tools, and database management systems that assessment developers use for finding and evaluating information. Use of tools, with auditing capability, that are web accessible, support asynchronous collaboration, and use computationally intelligent approaches are required to meet the demand for efficient and rapid development of systematic reviews (SR) and systematic evidence maps (SEMs) (Thayer et al. 2022, 2023). While these tools currently lack full automation and require a human in the loop, up and coming artificial intelligence (AI) move us toward it. The expectation is that moving toward automation using AI models will help address the increasing demand to rapidly extract the information informing assessments while adhering to foundational systematic (evidence mapping) methods. The workflows could be scalable and include the elasticity needed to rapidly scale (up or down) and reuse previous work in alignment with FAIR principles (Findable, Accessible, Interoperable, Reusable) (Wilkinson et al. 2016).

To date, the machine learning (ML)-assisted workflows implemented with SR tools and similar applications have principally eased the human burden by decreasing the amount of time spent screening studies at the title and abstract (TiAB) level, resulting in a significant reduction in overall time and costs associated with screening as reviewed by Van der Mierden et al. (2019). While these tools can facilitate the screening phase, they are not yet capable of automating the entire process, which includes complex tasks like data extraction and evidence synthesis. Therefore, summarizing the information available for decisions is still a challenge because existing tools still heavily rely upon manual user manipulation at each point in the workflow. Further, assessment teams in general lack software developers and therefore the coding knowledge required to write applications related to any form of large language model processing/AI. There are some tools with user interfaces that require little to no coding knowledge, but interoperability gaps exist meaning that coding knowledge is still required to migrate data between tool APIs. These gaps highlight further the institutional barriers to acquiring the needed infrastructure for hosting all the information technology (IT) components to fully enable intelligent document and data processing workflows. Therefore, assessment teams must partner with coding experts and their institutions to develop an ecosystem for developing the requirements needed for an intelligent document processing workflow, while providing training data needed by coders to refine ML models.

Developing ML models for literature screening and data extraction poses several additional challenges. First, research involves vast and diverse literature that spans multiple scientific disciplines, making it challenging to identify and extract relevant information. Second, many studies use complex methodologies, technical jargon, and highly specialized terminology, which can be difficult for ML algorithms to comprehend. Additionally, the lack of standardized language and terminology used in research can hinder the development of accurate ML models. Third, the quality of the available data can be highly variable, and the data may be biased or incomplete, which can lead to inaccurate results and therefore models. Fourth, the interpretation of results often requires a high level of domain expertise, making it challenging to validate the performance of ML models by human experts. Finally, ethical considerations, such as ensuring privacy and protecting sensitive information, must be carefully considered when developing ML models for any field. Addressing these challenges will require the integration of domain expertise, data quality control, and a collaborative effort between computer scientists and environmental health researchers to develop robust and reliable ML models for literature screening and data extraction in environmental chemical research.

In this paper, we describe a case application of AI/ML methods (i.e., incorporating them into new or existing applications) in human health environmental chemical assessments. Data extraction is currently one of the most laborious steps in the workflow and typically conducted by a PhD level scientist. Therefore, our objective is exploratory as we seek to understand the usability of a new data extraction tool (Dexter) https://pubmed.ncbi.nlm.nih.gov/34920276/, a web-based data extraction tool that provides a user-verification workflow of ML predictions for data entities in a chemical assessment workflow. In this exploratory case application, the goal is not to optimize model performance, but to understand if the data extraction experience is improved with ML models (assisted by humans) compared to a human only data extraction approach.

## 2. Methods

This methodology focuses on the data extraction component of a chemical assessment workflow (see Figure 1). This data extraction module will be used to conduct an evidence map based on the template methods for a Provisional Peer-Reviewed Toxicity Value (PPRTV) as described in U.S. EPA (2020) and in accordance with the systematic review methods used to conduct the SEMs as outlined in the Office of Research and Development (ORD) Staff Handbook for Developing Integrated Risk Information (IRIS) Assessments (U.S. EPA 2022) (IRIS Handbook). The specific methods developing the SEM, including database searches, and collecting references, the Populations Exposure, Comparator, and Outcome (PECO) criteria, and literature screening and tagging are described in the Supplemental Material, Appendices A-D. The aim of this study is to understand the usability of a new semi-automated data extraction tool, Dexter, in a chemical assessment workflow. Dexter is a web-based, customized version of the Laser AI tool (https://laser.ai), a systematic review tool based on AI. Dexter includes features that allow creation of customized data extraction form templates with features such as ML models for data extraction of animal and human health studies, with the additional options to include data extraction fields based on flat or hierarchical vocabularies (picklists), and to relate data elements to each other. The ML models underlying the data extraction in Dexter were originally developed as part of NIST-TAC SRIE as described by Nowak and Kunstman (2018) and Schmitt et al. (2018) with the additional Dexter features as described by Walker et al. (2022).

### 2.1 Semi-automated data extraction

#### 2.1.1 Data extraction strategy

**Figure 2.**
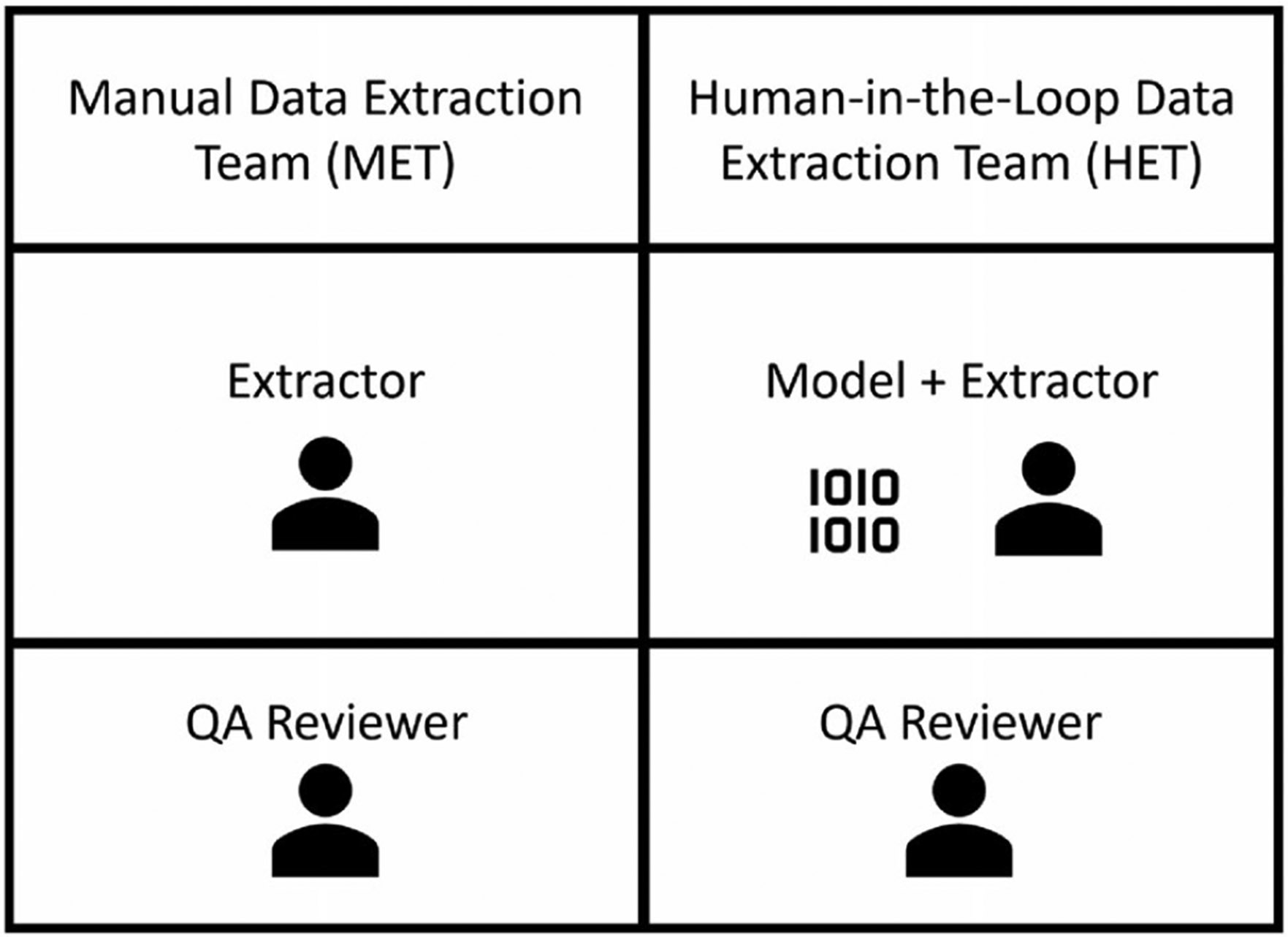
Data Extraction Teams. Two data extractors will independently extract data from references into forms using Dextr. The manual data extraction (ME) will manually extract all information while the semi-automated data extraction (SE) will manually extract all non-automated fields, while reviewing and correcting the model-generated extraction. Two different quality assurance (QA) reviewers will then correct both results for a given study to ensure accuracy of data extractions and calculate quality metrics.

The data extraction module of the workflow will be divided into two study arms: manual data extraction (ME) and semi-automated data extraction (SE) as depicted in Figure 2. The ME arm is designed to resemble the current review process with data extracted by one team member and QC by a different team member, as outlined in the IRIS Handbook. In the ME arm, one of the data extractors reads each study and manually extracts text from the methods section into the data extraction fields (see Appendix E, Supplementary Tables 1 and 2). In the SE arm, one data extractor reviews the model-generated data extractions and highlights. The data extractor makes corrections if needed to the extracted data, adding missing or removing extractions to fields as needed.

Both the ME and SE arms are implemented in Dexter using the fields described in Appendix E, for animal toxicology studies (Supplemental Table 7) or human health studies (Supplemental Table 8). The difference between the two arms and project set-up in Dexter is that there are no ML models attached to the ME data extraction fields during project set-up. The manual data extractors (ME arm) are also told to manually highlight the relevant extracted text in the PDF during the extraction process to not only facilitate QC, but also collect annotations that could be used for developing training data sets. Since the ML models in Dexter were previously trained exclusively from the methods sections of open access literature, the data extractors were instructed to restrict data extraction fromed to the methods sections only.

The results of extractions from ME and SE are then reviewed by two different quality assurance (QA) reviewers. The QA reviewers will be blinded regarding study arm allocation.

To control for differences between extractors' performance, the same extractors participate both in ME and SE study arms. For each extractor, the references assigned to them will be randomized across ME and SE in a 1:1 ratio. No extractor will extract or review the same reference across both arms. Each study arm has a separate Dextr project with identical data extraction forms and fields with picklists enabled. The ME project will not have models attached to the fields in the data extraction forms. To reduce variability associated with different levels of experience with the tool, all reviewers will complete one hour training on how to use the tool and will be required to perform extraction of two studies prior to starting the work. These two studies will not be included in the metrics for data extraction or reviewer time. Team members are instructed to not remain idle while signed into Dextr to ensure that accurate recording of the time to complete extractions is logged (Dexter automatically records the time users take to complete tasks). The times between start and stop actions will be manually checked against the event log in Dexter to ensure there were no cases of an extraction task being left idle, inflating the overall extraction time. Additionally, the user action logs from Dextr will be filtered for any periods of inactivity longer than 5 minutes and excluded from the calculation of time metrics. The data extractions will be exported from Dexter using the export feature. The HAWC client is used to import the datasets into the HAWC project (HAWC 2023). All data will be available for download.

### 2.2 Evaluation of semi-automated data extraction in the SEM workflow

The aim of the evaluation will be to understand how using the tool Dextr will perform as a semi-automated data extraction tool in an assessment workflow. The primary goals are to understand if the overall Dexter user experience was favorable and if the SE arm performed equal to or better than the ME arm. The evaluation will focus on the first pass data extraction, prior to QA results will not be incorporated into the first pass model performance metrics due to the potential to introduce complexity in resolving disagreements between reviewers.

First pass model performance will be evaluated manually and based on human review of the primary results from Dexter. A collection of metrics that include recall, precision, and F-1 score from selected data extraction fields from animal (test article name, species, strain, sex, and endpoint) or human (study population source, chemical name, exposure measurement type) will be evaluated initially. Results will be calculated by comparing the extracted data after primary extraction within each data extraction arm. Precision is the number of correct positive predictions made and is represented by the true positives/(true positives + false positives). Recall quantifies the number of correct positives made out of all correct positives that could have been made and is represented by true positives/(true positives + false negatives). The precision and recall score will be combined into a single F measure (the harmonic mean or F1 score) for summarizing overall model performance within a field. The F-measure will be calculated as (2*precision*recall)/(precision + recall).

The extracted data elements will be evaluated within an experimental arm for the number of correct data points entered for a specific field (i.e. test article name, species, endpoint name) from the primary extraction among all the primary extraction data points within a field. A data point will be marked as a “true positive” if it matches with the same element in the primary extraction + QA (i.e. when the human does not change the model response). A data point will be marked as a “false positive” if the element is included in the primary extraction by the model, but not included in the primary extraction + QA (i.e. a human makes a change to the model response). A data point will be marked as a “false negative” if the element is missed by the model in the SE arm and included by human in the manual extraction. A data point will be marked as a true negative when the model and the human do not fill in a field. True negatives will be identified by comparing data extracts to gold standard data available within HAWC (ORD SEM PFAS 150 (2022) ∣ HAWC (epa.gov) and https://hawc.epa.gov/assessment/100500328/).

The time metrics will be calculated both for the primary extraction and QA steps from both arms to measure the total extraction time for comparing the manual vs semi-automated arms. Total time for both the primary extraction as well as the time for QA will be evaluated.

### 2.3 Usability feedback

After completing the data extraction, user experience will be qualitatively evaluated through questionnaire (Appendix F, Supplemental Table 9).

### 2.4 Data storage

All data generated by the workflow describe here will be stored in HAWC and HERO at https://hawc.epa.gov/assessment/100500328/ and https://heronet.epa.gov/heronet/index.cfm/project/page/project_id/3591, respectively.

### 2.5 Caveats and limitations

This study will include extraction of 20 papers that have previously been extracted for comparison purposes as the primary intent is to evaluate the workflow (versus evaluation of model performance). Due to the novelty of the application, plausible estimates of the effect values across time and accuracy metrics are unknown, therefore formal sample size calculations are not provided, but are based on prior user and developer experience with Dexter.

The study is designed to inform US EPA application of semi-automated extraction tools. Hence, the workflow and data extraction forms resemble the ones used at US EPA. These conditions may not apply elsewhere, so the study results may not generalize to all settings in which literature reviews of environmental agents are performed.

Although not the primary goal of the current protocol, the manually curated results can be incorporated back into the models to refine the model's performance and then redeployed in Dextr. In subsequent projects, the new model performance can be reevaluated. Over time, it is expected that model performance will improve given that more data are available for training. The sustainable workflow is explicitly intended to include a human such that the workflow is semi-automated and model performance can be evaluated and improved with routine use of ML-facilitated data extraction over time.

## Supplementary Material

Supplement1

## Data Availability

The data and/or code that support this work are will be made available in HAWC (build 0b3967cd) and HERO (last updated April 3, 2024) at https://hawc.epa.gov/assessment/100500328/, ORD SEM PFAS 150 (2022) ∣ HAWC (epa.gov), https://hawc.epa.gov/assessment/100500328/, and https://heronet.epa.gov/heronet/index.cfm/project/page/project_id/3591, respectively. The Environmental Health Vocabulary is available at: https://hawc.epa.gov/vocab/ehv/. 3rd Party Tools including the software applications Dextr (LaserAI beta version 1.1.0), SWIFT Review (V1.43), and SWIFT Active Screener (V1.061.0794) are licensed applications.
