# Supplementary material for: Proof-of-concept for using machine learning to facilitate data extraction for human health chemical assessments: a study protocol
Source: Evid Based Toxicol. Author manuscript; Available in PMC 2025 Nov 11. (PMC12004489; doi:10.1080/2833373x.2024.2421192)
Supplement: Supplement1 [file NIHMS2060558-supplement-Supplement1.docx]

## Supplemental Materials: Literature Search, Screening, and Tagging Criteria

## Appendix A: Literature Search

### Database Search Term Development

The literature search focuses only on chemical name (and synonyms) with no additional limits.  Chemical synonyms are identified through [EPA’s CompTox Chemicals Dashboard](https://comptox.epa.gov/dashboard/). A chemical search using the CAS registration number and any synonyms indicated as “valid” or “good” were included in the search strings . The PubMed search is shared with information specialists to develop search strategies tailored for each of the databases below because each database has its own search syntax.

Literature search updates will be performed periodically during draft development, with the last full literature search update conducted within six months prior to the planned release of the finalized document. The results of the last literature update will screened against the PECO criteria and citation information available in HERO (see HERO description below). Newly identified studies are only incorporated into the assessment if they impact toxicity values.

### Database Searches and Filtering for Human Health Records

An information specialist searched PubMed, Web of Science, and Scopus through May 2023, and the results were stored and are maintained in the Health and Environmental Research Online (HERO) database [1,3-dinitrobenzene](https://heronet.epa.gov/heronet/index.cfm/project/page/project_id/3591).  The HERO database is used to provide access to the references used in US EPA’s scientific assessments, including this effort. Full details of the search strategy are presented in Supplemental Table 3. The search will be updated during the conduct of the case application with the last pre-manuscript submission update occurring ~ one month ahead of anticipated submission.

The records returned undergo deduplication in HERO using unique identifiers (e.g., PMID, WoSID, or DOI) and citation content. Following deduplication of the records, SWIFT-Review software (<https://www.sciome.com/swift-review/searchstrategies/>) is used to prioritize references for screening, based on use of the pre-set literature search strategies (“filters”): “animal (human health models)”, “human”, and “in vitro”. These strategies have been developed by information specialists and can be applied to distinguish studies containing human health-relevant information from studies with less relevant content (e.g., environmental fate).

Studies not retrieved or prioritized using the search strategies are not considered further. Studies that include one or more of the search terms in the title, abstract, keyword, or medical subject headings (MeSH) fields are exported as a RIS file for screening as described below.

Supplemental Table 1. Literature Search Strategies and Key Words

| **Databases** | **Search String** | | **Results** |
| --- | --- | --- | --- |
| WoS | TS=(“99-65-0” OR “1,3-dinitrobenzene”  OR “1,3-dinitrobenceno” OR “1,3-dinitrobenzol” OR “2,4-dinitrobenzene”  OR “3-dinitrobenzene” OR “benzene, 1,3-dinitro-“ OR “benzene, m-dinitro-“ OR “dinitrobenzene” OR “m-dinitrobenzene” OR “meta-dinitrobenzene”  OR “NSC 7189”) | | 3,574  (May 2023) |
| PubMed | (“99-65-0”[tw] OR “1,3-dinitrobenzene” [tw] OR “1,3-dinitrobenceno”[tw] OR “1,3-dinitrobenzol”[tw] OR “2,4-dinitrobenzene” [tw] OR “3-dinitrobenzene”[tw] OR “benzene, 1,3-dinitro-“[tw] OR “benzene, m-dinitro-“[tw] OR “dinitrobenzene”[tw] OR “m-dinitrobenzene”[tw] OR “meta-dinitrobenzene” [tw] OR “NSC 7189”[tw]) | | 2,895  (May 2023) |
| Scopus | TITLE-ABS-KEY({99-65-0} OR “1,3-dinitrobenzene”  OR “1,3-dinitrobenceno” OR “1,3-dinitrobenzol” OR “2,4-dinitrobenzene”  OR “3-dinitrobenzene” OR “benzene, 1,3-dinitro-“ OR “benzene, m-dinitro-“ OR {dinitrobenzene} OR “m-dinitrobenzene” OR “meta-dinitrobenzene”  OR {NSC 7189}) | | 10,562  (May 2023) |
| ***Unique records found* *from WoS, PubMed, and Scopus*** | | | **11,222** |
| ***Unique records from WoS, PubMed, and Scopus after application of filters in SWIFT Review for human, animal (human health), and in vitro evidence*** | | | **TBD** |
| ***Unique records identified from other resources*** | | | **TBD** |
| ***Total number of unique records screened at title and abstract level*** | | | **TBD** |
| Keyword highlighting utilized in SWIFT Active to facilitate manual (human) title and abstract screening: | | | |
| **Inclusion Keywords (green highlight)** | | **Exclusion Keywords (red highlight)** | |
| 1,3-Dinitrobenzene | | dinitrochlorobenzene | |
| 99-65-0 | | thioredoxin reductase | |
| 1,3-dinitrobenzene | | DNCB | |
| 1,3-dinitrobenceno | | 1-chloro-2,4-dinitrobenzene | |
| 1,3-dinitrobenzol | | 1-chloro | |
| 2,4-dinitrobenzene | | dinitrofluorobenzene | |
| 3-dinitrobenzene | | DNFB | |
| benzene, 1,3-dinitro | | chloro-2, 4- | |
| benzene, m-dinitro | | glutathione S-transferase | |
| dinitrobenzene | | glutathione | |
| m-dinitrobenzene | | S-transferase | |
| meta-dinitrobenzene | | difluoro | |
| NSC 7189 | | fluoro | |
| m-dinitrobenzene | | sulfonic | |
|  | | sulphonic acid | |
|  | | DNBS | |
|  | | sulfonate | |
|  | | o-dinitrobenzene | |
|  | | p-dinitrobenzene | |

### Other Resources Consulted for Primary Research Reports

The literature search strategies described above are designed to be broad, but like any search strategy, studies may be missed (e.g., cases where the specific chemical is not mentioned in title, abstract, or keyword content; ability to capture “gray” literature that is not indexed in the databases listed above). Thus, in addition to the database searches, the resources in Supplemental Table 4 will be used to identify studies that may have been missed based on the database search. These sources will be searched using customized processes described (Supplemental Table 3). References that appear to meet the PECO criteria will be uploaded into the screening software, annotated with respect to source of the record, and screened as described below according to PECO. Searching of these sources is summarized to include the source type or name, the search string (when applicable), the URL (when available and applicable), number of results, and number of unique references not otherwise identified from database searching. To identify unique references, a citation for each identified study is generated in HERO and verified that it is not already identified from the database searches (e.g., PubMed, WoS, etc) prior to moving forward to screening. Note, the Defense Technical Information Center (DTIC) is searched only for compounds pertinent to the Department of Defense. Therefore, 1,3-dintrobenzene was considered DOD-relevant due to its use in making explosives.

Supplemental Table 2. Grey Literature Sources for Primary Research Reports

| **Resource** | **Comments** |
| --- | --- |
| Bibliographies of included studies (studies meeting PECO) | Manual review (at the title/abstract) level of reference list in studies screened as PECO-relevant after full-text review. |
| Reference list from human health focused assessments | Review of the reference list from final or publicly available draft or finalized assessments (e.g., EPA IRIS [Integrated Risk Information System], ATSDR [Agency for Toxic Substances and Disease Registry] Toxicological Profile) or from published journal review specifically focused on human health. Reviews and assessments can be identified from the database search or surveys for existing assessments (Table 4). |
| References identified by technical consultants or during peer-review |  |
| AEGLs | Acute Exposure Guideline Levels for Airborne Chemicals (AEGL)  <https://www.epa.gov/aegl/access-acute-exposure-guideline-levels-aegls-values#chemicals> |
| AICIS | <https://services.industrialchemicals.gov.au/search-assessments/> |
| ChemView | EPA [ChemView](https://chemview.epa.gov/chemview) database to identify unpublished studies, information submitted to EPA under Toxic Substances Control Act (TSCA) Section 4 (chemical testing results), Section 8(d) (health and safety studies), Section 8(e) (substantial risk of injury to health or the environment notices), and FYI (For Your Information, voluntary documents). Other databases accessible via ChemView include EPA’s High Production Volume (HPV) Challenge database and the Toxic Release Inventory database. |
| DTIC | Defense Technical Information Center. Searched if topic is pertinent to the Department of Defense |
| ECETOC | [http://www.ecetoc.org/publications](https://gcc02.safelinks.protection.outlook.com/?url=http%3A%2F%2Fwww.ecetoc.org%2Fpublications&data=05%7C01%7CPersad.Amanda%40epa.gov%7Cdc06eae3ad6b401225cb08daf50e3add%7C88b378b367484867acf976aacbeca6a7%7C0%7C0%7C638091738710712183%7CUnknown%7CTWFpbGZsb3d8eyJWIjoiMC4wLjAwMDAiLCJQIjoiV2luMzIiLCJBTiI6Ik1haWwiLCJXVCI6Mn0%3D%7C3000%7C%7C%7C&sdata=XQe7EWkoDZeOMgoZkEC%2Bgafv%2BVbLcvkkOKN7cilk8r4%3D&reserved=0) |
| ECHA | European Chemicals Agency (ECHA) registration dossiers to identify data submitted by registrants. Registration dossiers contain data on substances such as hazardous properties, safe uses, classifications, environmental fate, and ecotoxicological and toxicological information. The amount of information provided for each substance varies and is obtained directly from companies’ REACH registrations. ECHA does not give any guarantees or warranties regarding the quality and correctness of the published information. The information in the portal is published ‘as provided’ by industry, and its accuracy has not been verified by ECHA (<https://echa.europa.eu/information-on-chemicals/registered-substances>). |
| JECDB | [http://dra4.nihs.go.jp/mhlw_data/jsp/SearchPageENG.jsp](https://gcc02.safelinks.protection.outlook.com/?url=http%3A%2F%2Fdra4.nihs.go.jp%2Fmhlw_data%2Fjsp%2FSearchPageENG.jsp&data=05%7C01%7CPersad.Amanda%40epa.gov%7Cdc06eae3ad6b401225cb08daf50e3add%7C88b378b367484867acf976aacbeca6a7%7C0%7C0%7C638091738710712183%7CUnknown%7CTWFpbGZsb3d8eyJWIjoiMC4wLjAwMDAiLCJQIjoiV2luMzIiLCJBTiI6Ik1haWwiLCJXVCI6Mn0%3D%7C3000%7C%7C%7C&sdata=j5iJqnNvApAu%2BaXiqbRGlLRiXsqIbu6mbxBMTbzquyc%3D&reserved=0) |
| NTP | National Toxicology Program (NTP) toxicology testing results and literature analysis reports. <https://ntp.niehs.nih.gov/> |
| NTRL | [https://ntrl.ntis.gov/NTRL/](https://gcc02.safelinks.protection.outlook.com/?url=https%3A%2F%2Fntrl.ntis.gov%2FNTRL%2F&data=05%7C01%7CPersad.Amanda%40epa.gov%7Cdc06eae3ad6b401225cb08daf50e3add%7C88b378b367484867acf976aacbeca6a7%7C0%7C0%7C638091738710712183%7CUnknown%7CTWFpbGZsb3d8eyJWIjoiMC4wLjAwMDAiLCJQIjoiV2luMzIiLCJBTiI6Ik1haWwiLCJXVCI6Mn0%3D%7C3000%7C%7C%7C&sdata=6MhODejRUl5oxnRThB%2B6ifUWyJCqXMOvvG9%2FTCYZ6xQ%3D&reserved=0) |
| OECD | [http://webnet.oecd.org/hpv/ui/Search.aspx](https://gcc02.safelinks.protection.outlook.com/?url=http%3A%2F%2Fwebnet.oecd.org%2Fhpv%2Fui%2FSearch.aspx&data=05%7C01%7CPersad.Amanda%40epa.gov%7Cdc06eae3ad6b401225cb08daf50e3add%7C88b378b367484867acf976aacbeca6a7%7C0%7C0%7C638091738710712183%7CUnknown%7CTWFpbGZsb3d8eyJWIjoiMC4wLjAwMDAiLCJQIjoiV2luMzIiLCJBTiI6Ik1haWwiLCJXVCI6Mn0%3D%7C3000%7C%7C%7C&sdata=9hYLe7balT4kBtPB2eiCoOkKTsxJ%2FvzUAI9DwC3ANK4%3D&reserved=0)  The Organisation for Economic Cooperation and Development (OECD) eChemPortal to retrieve results for OECD Screening Information DataSet (SIDS) and High Production Volume (HPV) Chemicals (<https://www.echemportal.org/echemportal/>). |
| US EPA’s CompTox Chemicals Dashboard ToxValDB | References from US EPA’s CompTox Chemicals Dashboard ToxValDB (Toxicity Values Database) to identify studies or assessments that present point of departure (POD) information. ToxValDB collates publicly available toxicity dose-effect related summary values typically used in risk assessments. Many of the PODs presented in ToxValDB are based on gray literature studies or assessments not available in databases such as PubMed, WoS, etc. It is important to note that ToxValDB entries have not undergone quality control to ensure accuracy or completeness and may not include recent studies. |
| EFSA | [http://www.efsa.europa.eu/](https://gcc02.safelinks.protection.outlook.com/?url=http%3A%2F%2Fwww.efsa.europa.eu%2F&data=05%7C01%7CPersad.Amanda%40epa.gov%7Cdc06eae3ad6b401225cb08daf50e3add%7C88b378b367484867acf976aacbeca6a7%7C0%7C0%7C638091738710712183%7CUnknown%7CTWFpbGZsb3d8eyJWIjoiMC4wLjAwMDAiLCJQIjoiV2luMzIiLCJBTiI6Ik1haWwiLCJXVCI6Mn0%3D%7C3000%7C%7C%7C&sdata=2J9TjKrdZwsfD50ec4h41OE2vfkwXErfs3TmKQIJgxs%3D&reserved=0) |

### Survey of Previous Assessments, Regulatory Reference Values, Risk Thresholds or Assessment Based Points of Departure

“Toxicity value” is a broad term that encompasses reference values and cancer risk estimates (i.e., slope factors and unit risk estimates). The term reference value applies to values designed to provide a “benchmark” or exposure limit below which adverse effects on human health are not expected to occur. Reference values are the most common final output from the dose response assessment component of the risk assessment paradigm set forth by the National Research Council and are based on an observed or estimated threshold for an effect, usually noncancer. Supplemental Table 3 presents a list of organizations that disseminate toxicity values queried to conduct a survey.

Supplemental Table 3. Sources Searched for Existing Human Health Reference Values and Cancer Descriptors

| **Source** | **Query and/or link** |
| --- | --- |
| ACGIH | ACGIH. 2007. 2007 TLVs and BEIs: Based on documentation of the threshold limit values for chemical substances and physical agents and biological exposure indices. Cincinnati, OH: American Conference of Governmental Industrial Hygienists. |
| AIHA | AIHA. 2019. 2019 ERPG/WEEL Handbook. Fairfax, VA: American Industrial Hygiene Association. [List of values.]  AIHA. 2002 (and updates). 2002 Emergency Response Planning Guidelines. Fairfax, VA: American Industrial Hygiene Association. [Details used in deriving values.] |
| ATSDR | <http://www.atsdr.cdc.gov/toxprofiles/index.asp> |
|  | https://wwwn.cdc.gov/TSP/MRLS/mrlsListing.aspx |
| US EPA’s CompTox Chemicals Dashboard ToxValDB | <https://comptox.epa.gov/dashboard>  *Note: ToxValDB collates publicly available toxicity dose-effect related summary values typically used in chemical assessments.  Many of the PODs presented in ToxVal are based on gray literature studies or assessments not available in databases such as PubMed and Web of Science (WoS).  Although many of the resources included in this table are represented in ToxValDB, they are also manually searched because most of the ToxValDB entries have not undergone quality control to ensure accuracy or completeness and might not include recent studies*. |
| CT DEEP | [https://eregulations.ct.gov/eRegsPortal/Browse/getDocument?guid={00D6A654-0300-CC47-9B95-397D2AD21304}](https://eregulations.ct.gov/eRegsPortal/Browse/getDocument?guid=%7b00D6A654-0300-CC47-9B95-397D2AD21304%7d) |
| DFG | <https://onlinelibrary.wiley.com/doi/10.1002/9783527826889.oth> |
| OW (US EPA) | https://www.epa.gov/system/files/documents/2022-01/dwtable2018.pdf |
|  | <https://www.epa.gov/dwstandardsregulations/drinking-water-contaminant-human-health-effects-information#dw-standards> |
| EPA/NRC AEGL | <https://www.epa.gov/aegl/access-acute-exposure-guideline-levels-aegls-values#chemicals> |
| European Commission | <https://eur-lex.europa.eu/legal-content/EN/TXT/PDF/?uri=CELEX:32017L0164&from=EN> |
| Health Canada | <https://www.canada.ca/en/services/health/publications/healthy-living.html> |
|  | <https://publications.gc.ca/collections/collection_2021/sc-hc/H129-108-2021-eng.pdf> |
|  | <http://publications.gc.ca/site/archivee-archived.html?url=http://publications.gc.ca/collections/Collection/H46-2-96-194E.pdf> |
| HEAST | <https://epa-heast.ornl.gov/heast.php> |
|  | <https://cfpub.epa.gov/ncea/risk/hhra/recordisplay.cfm?deid=2877> |
| HSA | <https://www.hsa.ie/eng/publications_and_forms/publications/chemical_and_hazardous_substances/chemical_agents_and_carcinogens_code_of_practice_2021.html> |
| IARC | <https://monographs.iarc.who.int/monographs-available/> |
|  | <http://monographs.iarc.fr/ENG/Classification/List_of_Classifications.pdf> |
| IDEM | <https://www.in.gov/idem/toxic/2343.htm> |
| ID DEQ | <https://adminrules.idaho.gov/rules/current/58/580101.pdf> |
| IFA | <https://limitvalue.ifa.dguv.de/WebForm_gw2.aspx> |
| IPCS | <https://www.inchem.org/pages/cicads.html> |
| IRIS | <http://www.epa.gov/iris/> |
| JSOH | <https://www.sanei.or.jp/?mode=view&cid=328> |
| MassDEP | <https://www.mass.gov/service-details/massdep-ambient-air-toxics-guidelines> |
| MDH | <https://www.health.state.mn.us/communities/environment/risk/guidance/air/table.html> |
| MI EGLE | <https://www.michigan.gov/documents/deq/deq-rrd-chem-CleanupCriteriaTSD_527410_7.pdf> |
| NATICH | <https://nepis.epa.gov/Exe/ZyPDF.cgi/2000NS7S.PDF?Dockey=2000NS7S.PDF> |
| NC DEQ | <https://files.nc.gov/ncdeq/Air%20Quality/rules/rules/D1104.pdf> |
| NDEP | <https://ndep.nv.gov/resources/risk-assessment-and-toxicology-basic-comparison-levels> |
| NIOSH | <http://www.cdc.gov/niosh/npg/npgdcas.html> |
|  | <https://www.cdc.gov/niosh/pubs/criteria_date_desc_nopubnumbers.html> |
|  | <https://www.cdc.gov/niosh/idlh/intridl4.html> |
| NJ DEP | <https://dep.nj.gov/boss/risk-screening-tools/> |
| NYSDEC | https://www.dec.ny.gov/docs/remediation_hudson_pdf/techsuppdoc.pdf |
| OAQPS (US EPA) | [https://www.epa.gov/fera/dose response-assessment-assessing-health-risks-associated-exposure-hazardous-air-pollutants](https://www.epa.gov/fera/dose-response-assessment-assessing-health-risks-associated-exposure-hazardous-air-pollutants) |
| OEHHA | <http://www.oehha.ca.gov/tcdb/index.asp> |
| Ontario MOL | <https://www.labour.gov.on.ca/english/hs/pubs/oel_table.php> |
| OPP (US EPA) | https://iaspub.epa.gov/apex/pesticides/f?p=chemicalsearch:1 |
| OR DEQ | <https://secure.sos.state.or.us/oard/displayDivisionRules.action?selectedDivision=1556> |
| OSHA | <https://www.osha.gov/chemicaldata/> |
| PAC Database | <https://pacteels.pnnl.gov/#/> |
| PPRTV | <https://www.epa.gov/pprtv/provisional-peer-reviewed-toxicity-values-pprtvs-assessments> |
| Publications Quebec | <http://legisquebec.gouv.qc.ca/en/showdoc/cr/S-2.1,%20r.%2013?csi_scan_9222d36c6a354dc6=BO9xyrMZ+270UP3j0MGuOD0kZjgFAAAAXrM3HA==&bcsi_scan_filename=S-2.1,%20r.%2013&bcsi_scan_9222d36c6a354dc6=KXzmpPueuN0L1AjnJOB1Zerr85YMAAAAyhrPTg==&bcsi_scan_filename=S-2.1,%20r.%2013> |
| RI DEM | <http://www.dem.ri.gov/programs/benviron/air/pdf/airtoxgl.pdf> |
| RIVM | <https://www.rivm.nl/bibliotheek/rapporten/711701092.pdf> |
|  | <https://www.rivm.nl/bibliotheek/rapporten/609021044.pdf> |
|  | <https://www.rivm.nl/bibliotheek/rapporten/711701025.pdf> |
| Safe Work Australia | <https://www.safeworkaustralia.gov.au/exposure-standards#exposure-standards-in-australia> |
| SWCAA | <http://www.swcleanair.org> |
| TCEQ | <https://www.tceq.texas.gov/toxicology/dsd/final> |
|  | <https://www.tceq.texas.gov/remediation/trrp/trrppcls.html> |
| USAPHC | <https://phc.amedd.army.mil/topics/envirohealth/hrasm/Pages/TG230.aspx> |
| VT DEC | <https://dec.vermont.gov/sites/dec/files/aqc/laws-regs/documents/AQCD%20Regulations%20ADOPTED_Dec132018.pdf#page=127> |
| WAC | <https://apps.leg.wa.gov/WAC/default.aspx?cite=173-460-150> |
| WHO | <https://inchem.org/pages/ehc.html> |
| Worksafe | <https://worksafe.govt.nz/topic-and-industry/work-related-health/monitoring/exposure-standards-and-biological-exposure-indices/> |

ACGIH = American Conference of Governmental Industrial Hygienists; AEGL = Acute Exposure Guideline Levels; AIHA = American Industrial Hygiene Association; ATSDR = Agency for Toxic Substances and Disease Registry; BEI = biological exposure index; CT DEEP = Connecticut Department of Energy & Environmental Protection; DFG = Deutsche Forschungsgemeinschaft, German Research Foundation; EPA = Environmental Protection Agency; ERGP = emergency response planning guideline; HEAST = Health Effects Assessment Summary Tables; HSA = Health and Safety Authority; IARC = International Agency for Research on Cancer; IDEM = Indiana Department of Environmental Management; ID DEQ = Idaho Department of Environmental Quality; IFA = Institut für Arbeitsschutz, The Institute for Occupational Safety and Health; IPCS = International Programme on Chemical Safety; IRIS = Integrated Risk Information System; JSOH = Japan Society for Occupational Health; MassDEP = Massachusetts Department of Environmental Protection; MDH = Minnesota Department of Health; MI EGLE = Michigan Environment, Great Lakes & Energy; MOL = Ministry of Labour; NATICH = National Air Toxics Information Clearinghouse; NC DEQ = North Carolina Department of Environmental Quality; NDEP = Nevada Division of Environmental Protection; NIOSH = National Institute for Occupational Safety and Health; NJ DEP = New Jersey Department of Environmental Protection; NRC = National Research Council; NYSDEC = New York State Department of Environmental Conservation; OAQPS = Office of Air Quality Planning and Standards; OEHHA = California Office of Environmental Health Hazard Assessment; OEL = occupational exposure level; OPP = Office of Pesticide Programs; OR DEQ = Oregon Department of Environmental Quality; OSHA = Occupational Safety and Health Administration; OW = Office of Water; PAC = Protective Action Criteria; PPRTV = Provisional Peer-Reviewed Toxicity Value; RfD = reference dose; RI DEM = Rhode Island Department of Environmental Management; RIVM = Rijksinstituut voor Volksgezondheid en Milieu, The Netherlands Institute for Public Health and the Environment; RSL = regional screening level; SWCAA = Southwest Clean Air Association; TCEQ = Texas Commission on Environmental Quality; TEEL = temporary emergency exposure limit; TLV = threshold limit value; TWA = time-weighted average; USAPHC = United States Army Public Health Center; VT DEC = Vermont Department of Environmental Conservation; WAC = Washington Administrative Code; WEEL = workplace environment exposure level; WHO = World Health Organization.

## Appendix B. Process for searching and collecting evidence from other resources

Review of reference lists from existing assessments (final or publicly available draft), journal reviews articles and studies considered relevant to PECO based on full-text screening.

Review of the citation reference lists is typically done manually because they are not available in a file format (e.g., RIS) that permits uploading into screening software applications. Manual review entails scanning the title, study summary, or study details as presented in the resource for those that appear to meet the PECO criteria. Any records identified that were not identified from the other sources will be imported into HAWC and screened. For tracking assessments or reviews, the name of the source citation and the number of records imported into HAWC will be noted. The reference list of any study included in the literature inventory will be reviewed manually to identify titles that appeared relevant to the PECO criteria. These citations will be tracked in a spreadsheet, compared against the literature base to determine if they are unique to the project, and then will be added to HAWC to be screened for PECO relevance.

Acute Exposure Guideline Levels for Airborne Chemicals (AEGLs)

The AEGL database (<https://www.epa.gov/aegl/access-acute-exposure-guideline-levels-aegls-values#chemicals>) is searched by chemical name and CASRN. Any available reference value documentation is tracked.

Australian Industrial Chemicals Introduction Scheme (AICIS)

The AICIS assessment page (<https://services.industrialchemicals.gov.au/search-assessments/>) is searched by chemical CASRN.

Defense Technical Information Center (DTIC)

For Department of Defense-relevant chemicals, DTIC is searched by chemical name, synonyms, and CASRN, and the top 100 results are retrieved. Every record marked “APPROVED FOR PUBLIC RELEASE” that it is not already identified from the database search (or searches of “other sources consulted”) is screened at the title-and-abstract level. For those deemed potentially relevant, a citation is generated in HERO. References will then be further screened in HAWC against the PECO criteria.

ECETOC

The ECETOC publications list (<http://www.ecetoc.org/publications>) is searched by chemical name and CASRN. Any record that it is not already identified from the database search (or searches of “other sources consulted”) is uploaded to HAWC and screened against the PECO criteria.

EPA CompTox Chemicals Dashboard (ToxValDB)

ToxValDB is searched in the EPA CompTox Chemicals Dashboard , and data available from the Hazard tab is exported from the CompTox File Transfer Protocol site (<ftp://newftp.epa.gov/COMPTOX/STAFF/rjudson/datasets/ToxValDB/2019-08-20/>). Using both the human health POD summary file and the Record Source file, citations are identified that apply to human health PODs. A citation for each referenced study is generated in HERO and verified that it is not already identified from the database search (or searches of “other sources consulted”) prior to moving forward to screening in HAWC.

European Chemicals Agency (ECHA)

A search of the ECHA registered substances database is conducted using the CASRN. The registration dossier associated with the CASRN is retrieved by navigating to and clicking the eye-shaped view icon displayed in the chemical summary panel. The general information page and all subpages included under the Toxicological Information tab are downloaded in PDF (Portable Document Format), including all nested reports having unique URLs.

At this stage, each study summary is reviewed for inclusion based on PECO criteria. Study summaries identified as without administrative data information are excluded from review, and study summaries labeled “read across” (if any) are screened and considered supplemental material. When a study summary considered relevant reported data from a study or lab report, a citation for the full study is generated in HERO and verified that it is not already identified from the database search (or searches of “other sources consulted”) prior to moving forward to screening.

European Food Safety Authority (EFSA)

The EFSA online journal is searched by chemical name, synonyms, and CASRN. Any records returned that have not already been identified from the database search (or searches of “other sources consulted”) are added to HERO and screened in HAWC.

EPA ChemView

The EPA ChemView database is searched using the chemical CASRN. The prepopulated CASRN match and the “Information Submitted to EPA” output option filter are selected before generating results. If results are available, the square-shaped icon under the “Data Submitted to EPA” column is selected, and the following records are included:

- High Production Volume Challenge Database (HPVIS)
- Human Health studies (Substantial Risk Reports)
- TSCA Section 4 (Chemical testing results)
- TSCA Section 8(d) (Health and safety studies)
- TSCA Section 8(e) (Substantial risk)
- FYI (Voluntary documents)

All records for ecotoxicology and physical and chemical property entries are excluded. When results are available, extractors navigate into each record until a substantial risk report link is identified and saved as a PDF file. Substantial risk reports move forward to HAWC for screening according to PECO and supplemental material criteria.

Japan Existing Chemical Database (JECDB)

The database (<https://dra4.nihs.go.jp/mhlw_data/jsp/SearchPageENG.jsp>) is searched by chemical CASRN. References that look potentially relevant based on PECO criteria are tracked and screened in HAWC.

National Toxicology Program (NTP)

[Chemical Effects in Biological Systems (CEBS) database](https://manticore.niehs.nih.gov/cebssearch) of study results and research projects (<https://cebs.niehs.nih.gov/cebs/>) and literature assessment (<https://ntp.niehs.nih.gov/publications/index.html>). These resources can be searched by using the general search field for the primary chemical name or CASRN and selecting applicable filters (i.e., testing program, literature analysis).

The Chemical Effects in Biological Systems database is searched using the chemical CASRN (<https://manticore.niehs.nih.gov/cebssearch>). All non-NTP data are excluded using the “DTT Data Only” filter. Data tables for reports undergoing peer review (<https://ntp.niehs.nih.gov/data/tables/index.html>), the Report on Carcinogens ([https://ntp.niehs.nih.gov/whatwestudy/assessments/cancer/roc/index.html](https://gcc02.safelinks.protection.outlook.com/?url=https%3A%2F%2Fntp.niehs.nih.gov%2Fwhatwestudy%2Fassessments%2Fcancer%2Froc%2Findex.html&data=05%7C01%7CPersad.Amanda%40epa.gov%7Cdc06eae3ad6b401225cb08daf50e3add%7C88b378b367484867acf976aacbeca6a7%7C0%7C0%7C638091738710712183%7CUnknown%7CTWFpbGZsb3d8eyJWIjoiMC4wLjAwMDAiLCJQIjoiV2luMzIiLCJBTiI6Ik1haWwiLCJXVCI6Mn0%3D%7C3000%7C%7C%7C&sdata=5enOpb7sX6lHcWAappLYqCXoRAFrAr0VFPmL1prYbeI%3D&reserved=0)), and the NTP publication index (<https://ntp.niehs.nih.gov/publications/index.html>) are also searched by chemical name.

National Technical Reports Library (NTRL)

NTRL is searched by chemical name, synonyms, and CASRN. HERO citations are generated for the top 100 search results. Records not already identified from the database search (or searches of “other sources consulted”) are then uploaded to HAWC for screening. For NTRL, the first 100 records are screened, and then additional batches of 100 are screened if more than one PECO relevant reference is identified.

Organisation for Economic Co-operation and Development (OECD) Echem Portal

The OECD Echem Portal (<https://hpvchemicals.oecd.org/UI/Search.aspx>) is searched using the chemical CASRN. Only database entries from the following sources are included, and entries from all other databases are excluded in the search.

- OECD HPV
- OECD SIDS IUCLID
- SIDS United Nations Environment Programme (UNEP)

Final assessment reports and other relevant SIDS reports embedded in the links are captured and saved as PDF files.

## Appendix C: Populations, Exposures, Comparators, and Outcomes (PECO) Criteria and Supplemental Material Tagging

PECO criteria are used to focus the scope of an evidence map or systematic review by defining the research question(s), search terms, and inclusion/exclusion criteria. The PECO criteria for this case example is presented in Supplemental 4. In addition to PECO-relevant studies, studies that do not meet PECO criteria but contain “potentially relevant” supplemental material will be tracked during the literature screening process. Supplemental material will be tagged by category, as outlined in Supplemental 4.

Supplemental Table 4. Populations, Exposures, Comparators, and Outcomes (PECO) criteria

| PECO element | Evidence |
| --- | --- |
| Populations | Human: Any population and lifestage (occupational or general population, including children and other sensitive populations).  Animal: Nonhuman mammalian animal species (whole organism) of any lifestage (including fetal, early postnatal, adolescents and adults). |
| Exposures | **Relevant forms:**  “1,3-Dinitrobenzene”, "99-65-0" OR "1,3-dinitrobenzene", "1,3-dinitrobenceno", "1,3-dinitrobenzol", "2,4-dinitrobenzene", "3-dinitrobenzene", "benzene, 1,3-dinitro-", "benzene, m-dinitro-", "dinitrobenzene", "m-dinitrobenzene", "meta-dinitrobenzene", "NSC 7189"  Human: Any exposure to 1,3-dinitrobenzene via oral or inhalation routes. Citations will also be included if biomarkers of exposure are evaluated (e.g., measured chemical or metabolite levels in tissues or bodily fluids) but the exposure route is unclear or likely from multiple routes. Other exposure routes, such as those that are clearly dermal, will be tracked during title and abstract screening and tagged as “Supplemental – Non-PECO route of exposure.”  Animal: Any exposure to 1,3-dinitrobenzene via oral or inhalation routes of ≥14 d duration, or any duration assessing exposure during reproduction or development. Studies involving exposures to mixtures will be included only if they include an experimental arm with exposure to a defined mixture including the chemical of interest. Other exposure routes, including [dermal or injection], are tracked during title and abstract as “Supplemental – Non-PECO route of exposure.” |
| Comparators | **Human:** A comparison or referent population exposed to lower levels (or no exposure/exposure below detection limits), or exposure for shorter periods, or cases versus controls, or a repeated measures design. However, worker surveillance studies are considered to meet PECO criteria even if no statistical analyses using a referent group are presented. Case reports or case series of >3 people will be considered to meet PECO criteria, while case reports describing findings in 1–3 people will be tracked as “supplemental – Case reports or case series l.”  Animal: A concurrent control group exposed to vehicle-only treatment or untreated control (control could be a baseline measurement, e.g., acute toxicity studies of mortality, or a repeated measure design). |
| Outcomes | All health outcomes (cancer and noncancer). In general, endpoints related to clinical diagnostic criteria, disease outcomes, biochemical, histopathological examination, or other apical/phenotypic outcomes are considered to meet PECO criteria. Mechanistic data are tagged as “Supplemental – Mechanistic endpoints.” |

Supplemental Table 5. Categories of Supplemental Material in the PPRTV Program1

| **Category** | **Description** |
| --- | --- |
| **Pharmacokinetic**   - **ADME (absorption, distribution, metabolism, and excretion)** - **Classical pharmacokinetic (PK)** - **Physiologically based pharmacokinetic (PBPK) model studies** | The category of pharmacokinetic studies includes ADME (absorption, distribution, metabolism, and excretion) studies, classical pharmacokinetic (PK) or dosimetry model studies, and physiologically based pharmacokinetic (PBPK) or mechanistic dosimetry model studies.  ADME studies are primarily controlled experiments, where defined exposures usually occur by intravenous, oral, inhalation, or dermal routes, and the concentration of particles, a chemical, or its metabolites in blood or serum, other body tissues, or excreta are then measured. These data are used to estimate the amount absorbed (A), distributed (D), metabolized (M), or excreted (E). ADME data can also be collected from human subjects who have had environmental or workplace exposures that are not quantified or fully defined. ADME data, especially metabolism and tissue partition coefficient information, can be generated using in vitro model systems. Although in vitro data may not be as definitive as in vivo data, these studies should also be tracked as Pharmacokinetic.  Classical PK or dosimetry modeling usually divides the body into just one or two compartments, which are not specified by physiology, where movement of a chemical into, between, and out of the compartments is quantified empirically by fitting model parameters to ADME data.  PBPK models represent the body as various compartments (e.g., liver, lung, slowly perfused tissue, richly perfused tissue) to quantify the movement of chemicals or particles into and out of the body (compartments) by defined routes of exposure, metabolism, and excretion, and thereby estimate concentrations in blood or target tissues.  Pharmacokinetic studies are further subtagged as either in vivo, in vitro, or model.  *Studies describing environmental fate and transport or metabolism in bacteria or model systems that are not applicable to humans or animals are not considered relevant and should be excluded. |
| **Mechanistic endpoints** | Studies that do not meet PECO criteria but report measurements that inform the biological or chemical events associated with phenotypic effects related to a health outcome. Experimental design may include in vitro, in vivo (by various routes of exposure; includes all transgenic models), ex vivo, and in silico studies in mammalian and nonmammalian model systems. Studies using new approach methodologies (NAMs; e.g., high throughput testing strategies, read-across applications) are also categorized here. Studies where the chemical is used as a laboratory reagent (e.g., as a chemical probe used to measure antibody response) generally are not considered relevant and should be excluded.  For PPRTV assessments, genotoxicity mechanistic studies are specifically sub-tagged.  *Several chemicals that don’t meet PECO for 1,3-dinitrobenzene are commonly used as model haptens to explore aspects of mechanisms of immune function, allergenicity, and dermal sensitization [e.g., dinitrochlorobenzene (DNCB), dinitrofluorobenzene (DNFB)]. Abstract were excluded that described the use of a specific immune/allergenicity/dermal sensitization assay tested on reference compounds but did not specifically mention 1,3-dinitrobenzene. |
| **Non-PECO route of exposure** | Epidemiological or animal studies that use a non-PECO route of exposure, (e.g., injection studies or dermal studies if the dermal route is not part of the exposure criteria).  *This categorization generally does not apply to epidemiological studies where the exposure route is unclear; such studies are considered to meet PECO criteria if the relevant route(s) of exposure are plausible, with exposure being more thoroughly evaluated at later steps. |
| **Non-PECO exposure duration** | PPRTV assessments focus on subchronic and chronic exposure durations. Thus, short-term and acute exposure durations (defined as animal studies of less than 14 d in duration) are considered supplemental. |
| **Case reports or case series** | Human studies that present an investigation of a single exposed individual or group of ≤3 subjects that describe health outcomes after exposure but lack a comparison group (i.e., do not meet the “C” in the PECO criteria) and typically do not include reliable exposure estimates. |
| **Records with no original data** | Records that do not contain original data, such as other regulatory agency assessments, informative scientific literature reviews |
| ^1^This approach is a modification of the tagging scheme for supplemental material in EPA IRIS assessments {U.S. EPA, 2022, 10367891}. This scaled back tagging scheme reflects the data poor nature and reduced evidence base complexity of PPRTV assessments in comparison to IRIS assessments, e.g., PBPK modeling is not pursued in PPRTV assessments. | |

## Appendix D: Screening and Tagging Process

As described below, two different software applications will used for screening. Regardless of the software application, both TiAB and full-text screening are conducted by two independent reviewers and any conflicts in screening are resolved by discussion between the two independent reviewers; a third reviewer is consulted if any conflicts remained thereafter. Conflicts between screeners in applying the supplemental tags are resolved by discussion at both the TiAB and full-text levels, erring on the side of over-tagging at the TiAB level. At the TiAB level, articles without an abstract are screened based on title (title should indicate clear relevance) and number of pages (articles two pages in length or less are assumed to be abstracts or short communications rather than full study reports and tracked as supplemental).

The records identified from the evidence streams “animal (human health models)”, “human”, and “in vitro” in SWIFT-Review will be deduplicated by “Title” and imported into SWIFT-Active Screener, for TiAB screening. SWIFT-Active Screener is a web-based collaborative software application that utilizes active learning approaches to reduce the screening effort. The screening process is designed to prioritize records that appear to meet PECO criteria or include supplemental material content based on TiAB content (i.e., both types of records were screened as “include” for active learning purposes). Key words are used to facilitate the screening process (Supplemental Table 6). Records are screened in SWIFT-Active Screener at the TiAB level until the software indicates a likelihood of 95% that all relevant records had been captured. This threshold is comparable to human error rates and is used as a metric to evaluate machine-learning performance (Bannach-Brown et. al. 2018; Howard et al., 2016; Cohen et al., 2006). Any records in “partially screened” status at the time of reaching the 95% threshold are considered fully screened by accepting the predicted decision from the model.

All TiAB screening decisions (human and machine) made in SWIFT Active will be imported into US EPA’s version of the Health Assessment Workspace Collaborative (HAWC), a free and open source web-based software application that facilitates the management of assessments for environmental pollutants. The TiAB-specific tags are used to separate records meeting PECO criteria (which undergo full-text review in HAWC) versus supplemental content (Supplemental Table 2) and to tag certain specific categories of supplemental content (pharmacokinetic, genotoxicity, non-PECO route, non-PECO exposure duration, case reports or series, and records with no original data). For records meeting PECO criteria at the TiAB level, full text articles are retrieved through US EPA’s HERO database and linked to the record in HAWC. Most tagging of supplemental content occurs during TiAB screening, but tagging can also be done during full-text screening for studies that meet PECO criteria and also contain supplemental content. References that are not able to be retrieved within 45 days will not considered further. Records identified via the gray literature searches will be imported directly into HAWC where screening begins at the TiAB phase.

HAWC is used for full-text review. The full text articles found for records meeting PECO are available in HERO, which is linked to the record in HAWC. For full-text review, the methods and results sections from records are used to further determine PECO-relevance and to apply more tags to the record. Like TiAB level review, two reviewers independently determine inclusion with conflicts resolved in the same manner.

Supplemental Table 6. Keyword highlighting utilized in SWIFT Active (title and abstract screening) and HAWC (full text) to facilitate manual (human) screening

| **Inclusion Keywords (green highlight)** | **Exclusion Keywords (red highlight)** |
| --- | --- |
| 1,3-Dinitrobenzene | dinitrochlorobenzene |
| 99-65-0 | thioredoxin reductase |
| 1,3-dinitrobenzene | DNCB |
| 1,3-dinitrobenceno | 1-chloro-2,4-dinitrobenzene |
| 1,3-dinitrobenzol | 1-chloro |
| 2,4-dinitrobenzene | dinitrofluorobenzene |
| 3-dinitrobenzene | DNFB |
| benzene, 1,3-dinitro | chloro-2, 4- |
| benzene, m-dinitro | glutathione S-transferase |
| dinitrobenzene | glutathione |
| m-dinitrobenzene | S-transferase |
| meta-dinitrobenzene | difluoro |
| NSC 7189 | fluoro |
| m-dinitrobenzene | sulfonic |
|  | sulphonic acid |
|  | DNBS |
|  | sulfonate |
|  | o-dinitrobenzene |
|  | p-dinitrobenzene |

## Appendix E: Data Extraction Fields

Supplementary Table 7. Animal Data Extraction Fields

| **Domain/Field Na­­me** | **Picklist or free text** | **Help text** | **Model?** |
| --- | --- | --- | --- |
| **Test article** | **Domain heading** | **Domain heading** |  |
| Test article name | Free text | Select the chemical name (test material) as reported by authors and the appropriate link to chemical information (if available) from the CompTox Chemicals Dashboard. | Yes |
| ­­­CAS number | Free text | Select the appropriate CAS number. | Yes |
| Vehicle | Free text | Description of the vehicle (use name as described in methods but also add the common name if the vehicle was described in a non-standard way). | Yes |
| Route of exposure | Picklist  Oral Inhalation Dermal Subcutaneous  Intraperitoneal  Intravenous Other  Not reported | Description of the primary route of exposure. If multiple primary-exposures, select ‘Other’, highlight appropriate text, and provide details in the comments section. | Yes |
| Study | **Domain heading** |  |  |
| Experiment type | Picklist  Short-term (1-30 days)  Subchronic (30-90 days)  Chronic (>90 days)  Mechanistic  Reproductive  Developmental  Other  Acute (<24 hr) | Select experiment type as appropriate |  |
| **Animal model** | **Domain heading** | **Domain heading** |  |
| Species | Picklist | Select species as appropriate from the picklist. If not available from the picklist select ‘Other’, highlight appropriate text, and provide details in the comments section. | Yes |
| Strain | Picklist | Select strain as appropriate. If not available from the picklist select ‘Other’, highlight appropriate text, and provide details in the comments section. | Yes |
| Sex | Picklist  Male  Female  Combined  Not reported | Select sex as appropriate. | Yes |
| **Endpoint** | **Domain heading** | **Domain heading** |  |
| Endpoint name | Picklist  Available endpoint names can be found at https://hawc.epa.gov/vocab/ehv/ (Endpoint/Outcome column) | From the picklist select the relevant endpoint addressed by this study summary. | Yes |

Supplemental Table 8. Epidemiology Data Extraction Fields

| **Field** | **Choices** | **description** | **Semi-automated?** |
| --- | --- | --- | --- |
| **Study characteristics** | **Domain heading** | **Domain heading** |  |
| Study design | Picklist  cohort  case-control  RCT  Ecological  Cross-sectional  Other  Not reported | Select the most appropriate design from the list. If more than one study design applies (e.g., a cohort with cross-sectional analyses of baseline measures), can either a) select one design ("cohort") and clarify different timing in remaining extraction or b) select "other" and provide details in comments. | Yes |
| **Population** | **Domain heading** | **Domain heading** |  |
| Study population source | Picklist  Occupational  General population  Other | Describe the source of the study population. | No |
| Overall study population (N) | Free text | Describe numerically the number of participants in the study population | No |
| Population age category | Picklist  Adults  Children and adolescents 1-18 yrs  Pregnant women  Infants <1 yr  Other  Not reported | Select the most appropriate population age category from the list. | No |
| Population age details | Free Text | Describe the study population`s age category. | No |
| sex | Picklist  Male Female Male and Female  Not reported | Description of population sex | No |
| Country | Free Text | Describe the country of residence of the study population | No |
| Additional geographic information (optional) | Free text | Optional field, describe additional geographic information regarding the study population. | No |
| **Exposure** | **Domain heading** | **Domain heading** |  |
| Chemical name | Free text | This field is commonly used in visualizations, so consider using a common acronym, e.g., BPA instead of Bisphenol A | Yes |
| Exposure measurement type | Picklist  Biomonitoring  Blood (portion: Plasma)  Blood (portion: Whole blood)  Blood (portion: Serum)  Urine  Teeth  Nails  Hair  Saliva  Breast milk  Semen  Feces  Cerebrospinal fluid  Exhaled breath  Other  Air  Food  Drinking water  Occupational  Modeled  Questionnaire  Direct administration - oral  Direct administration - inhalation | Select the most appropriate type from the list. If a study includes multiples exposure measurement types but they are analyzed with outcomes separately, create a separate entry for each. If more than one type is combined for analysis with an outcome, you can select multiple options from the list. "Occupational" should be used when the exposure is based on job duties, etc. (i.e., not occupational exposure measured by biomarkers or air). | No |
| Exposure route | Picklist  Inhalation  Oral  Dermal  In utero  Intravenous  Unknown/Total  Other  Not reported | Description of the primary route of exposure. If multiple primary exposures, select ‘Other’, highlight appropriate text, and provide details in the comments section. | Yes |
| **Outcomes** | **Domain heading** | **Domain heading** |  |
| Outcome name | Picklist  See Supplemental Materials, Appendix E | A unique name for the specific outcome being measured. The endpoint is generally more specific than the effect (e.g., total cholesterol, incident asthma within the previous year, WISC-IV full scale). Use controlled vocabulary when available. If not available, enter outcome as free text. | Yes |

## Appendix F: Usability Questionnaire

Supplemental Table 9. Usability Feedback Questionnaire

| Question |
| --- |
| On a scale of 1 out of 10 with 10 representing the best experience, how would you rate the usability of Dexter? |
| How would you compare (pros and cons) of Dexter compared to other data extraction tools used by EPA (such as DistillerSR and EPA HAWC)? |
| What did you like most about the Dexter tool and why? |
| What did you like least about the Dexter tool and why? |
| What did you think about the user interface and navigation? |
| Is there anything that you would like to see in future versions of the tool? |
